# Exploring the effects of task shifting for HIV through a systems thinking lens: the case of Burkina Faso

**DOI:** 10.1186/1471-2458-13-997

**Published:** 2013-10-22

**Authors:** Fadima Yaya Bocoum, Seni Kouanda, Bocar Kouyaté, Sennen Hounton, Taghreed Adam

**Affiliations:** 1 Département biomédical et santé publique, Institut de Recherche en Science de la Santé, Ouagadougou, Burkina Faso; 2 University of Western Cape, School of Public Health, Cape Town, South Africa; 3 Ministry of Health, Ouagadougou, Burkina Faso; 4 Technical Division, United Nations Population Fund, New-York, USA; 5 Alliance for Health Policy and Systems Research, World Health Organization, Geneva 1211, Switzerland

**Keywords:** Task shifting, Task delegation, Evaluation, Systems thinking, Health systems research, System-wide impact

## Abstract

**Background:**

While the impact of task shifting on quality of care and clinical outcomes has been demonstrated in several studies, evidence on its impact on the health system as a whole is limited. This study has two main objectives. The first is to conceptualize the wider range of effects of task shifting through a systems thinking lens. The second is to explore these effects using task shifting for HIV in Burkina Faso as a case study.

**Methods:**

We used a case study approach, using qualitative research methods. Data sources included document reviews, reviews of available data and records, as well as interviews with key informants and health workers.

**Results:**

In addition to the traditional measures of impact of task shifting on health outcomes, our study identified 20 possible effects of the strategy on the system as a whole. Moreover, our analysis highlighted the importance of differentiating between two types of health systems effects. The first are effects inherent to the task shifting strategy itself, such as job satisfaction or better access to health services. The second are effects due to health system barriers, for example the unavailability of medicines and supplies, generating a series of effects on the various components of the health system, e.g., staff frustration.

Among the health systems effects that we found are positive, mostly unintended, effects and synergies such as increased health workers' sense of responsibility and worthiness, increased satisfaction due to using the newly acquired skills in other non-HIV tasks, as well as improved patient-provider relationships. Among the negative unintended effects are staff frustration due to lack of medicines and supplies or lack of the necessary infrastructure to be able to perform the new tasks.

**Conclusion:**

Our analysis highlights the importance of adopting a systems thinking approach in designing, implementing and evaluating health policies to mitigate some of the design issues or system bottle-necks that may impede their successful implementation or risk to present an incomplete or misleading picture of their impact.

## Background

According to the World Health Organization, at least 57 low and middle income countries (LMICs) are facing a critical shortage of health workforce, creating a major bottleneck for scaling up health services and achieving health systems goals [1]. In order to address this shortage, many LMICs have been implementing various forms of task-shifting strategies. Task shifting has been widely used in Sub Saharan Africa [2], covering a variety of health services such as Malaria control [3], cesarean section [4,5], HIV care and treatment [6] and coverage of maternal and child health services [7]. It is defined as the delegation of tasks to less-specialized cadres. For HIV care, it often includes enabling nurses to dispense antiretroviral therapy (ART) and capacitating community health workers to deliver a range of HIV services [8].

While several studies assessed the impact of task shifting, the majority focused on the effects of the strategy on quality of care or health outcomes [9,10]. Very few assessed its wider range of impact on the health system as a whole [11,12]. This observation is not unique to evaluations of task shifting strategies. A recent review of evaluations of health systems strengthening interventions in LMICs showed that while most of the evaluated interventions were complex, involving at least two or more health systems building blocks, less than half of the evaluations asked a broad set of research questions to allow for a wider assessment of the intervention's impact on the health system as a whole. Out of the seven evaluations of task shifting included in this review, only one explored the wider range of impact of the intervention on the health system [13]. Moreover, given the nature of complex adaptive systems, such as health systems, they are constantly changing and adapting in response to the introduction of new strategies and policies [14,15]. Considering the wider impact of health systems strengthening policies and strategies on health systems, therefore, requires a system thinking approach to be able to better understand how they work, for whom and under what circumstances, including their possible intended and unintended effects on the system itself as well as its actors and stakeholders [16].

This study aims to contribute to the increasing interest in employing systems thinking concepts in designing and evaluating complex health systems strengthening interventions and in exploring how this can be done in practice [16,17]. It does so through two main objectives. The first is to conceptualize the wider range of effects of task shifting through a systems thinking lens [13]. The second is to explore these effects using task shifting for HIV in Burkina Faso as a case study. Since the effects of task shifting on quality of care and health outcomes have been well documented in various countries [9,10], we listed these effects in our conceptual framework but only focused on the wider impact of task shifting on the health system as a whole in our data collection and analysis. Moreover, it is worth noting that our goal was not to perform a large-scale evaluation of task shifting for HIV in Burkina Faso using this framework –this is beyond the scope of this study. Instead, our goal is to explore how such thinking and comprehensive evaluations of system-wide effects can be conceptualized and applied in practice to learn from and guide future studies of this nature.

### Task shifting for HIV as conceived in Burkina Faso

With around 1.6% of the adult population in Burkina Faso infected with the HIV/AIDs virus in 2006 [18], the Ministry of Health together with several technical and financial partners decided to develop a task shifting strategy for HIV/AIDS to be delivered at different level of the health system, see Table 1. As part of the strategy, teams of health facility-based and community-based personnel were formed to ensure access to HIV services at peripheral level. Consequently, new collaborations were established with community-based organizations, by which community health workers were trained to provide some HIV services, as described in Table 1. Community health workers were given financial incentives for performing these tasks through funds granted by the Global Fund for HIV, Tuberculosis and Malaria as part of a larger HIV programme of work.

**Table 1:** Comparison of planned versus actual tasks shifted by cadre for providing HIV services in Burkina Faso

**Location of service/cadre**	**Tasks originally planned to be shifted or new tasks for other cadres**	**Tasks *not *** **implemented or shifted in practice**
**District hospital**		
New tasks for generalist doctors with or without HIV training*	Assessment of ART eligibility; ART initiation and follow up; hospitalization care if necessary; Management of ART side effects;	Management of ART side effects; Adherence support.
	Post exposure prophylaxis (PEP); Referral of complicated cases to regional or national hospital; Adherence support; Training, mentoring and supervision at primary health care centres.	
From doctors to Nurses/ midwives	Testing and clinical follow up for HIV and opportunistic infections; Adherence support; Management of ART side effects.	Adherence support.
New tasks for pharmacists	Follow up of adherence to ART; Management and refill of medicines (ART, OI, etc.); Training, mentoring and supervision of nurses at primary health care centres and drug shop managers in the catchment area.	Training, mentoring and supervision of nurses at primary health care centres and drug shop managers in the catchment area.
From laboratory technologists at district hospitals to nurses and midwives at primary care facilities	Laboratory tests.	
New tasks for social workers	Psychosocial support; Home visits; Search for lost to follow up; Social enquiry on PLWHA.	Psychosocial support; Home visits; Search for lost to follow up; Social enquiry on PLWHA.
**Health centres**		
From district hospital staff to nurses at primary health centres	VCT; Clinical follow up; Detection of TB; Management of common OI; Refer complicated cases to district or regional hospital; ART refill; Management of ART side effects;	Clinical follow up; Management of common OI; Refer complicated cases to district or regional hospital;
		ART refill; Management of ART side effects.
From nurses to auxiliary midwives	Promotion of prevention measures; Promotion and provision of VCT; PMTCT; Adherence support; Home visits; Search for lost to follow up.	Promotion of prevention measures; Promotion and provision of VCT;
		Adherence support; Home visits; Search for lost to follow up.
**Community based organizations**		
New tasks for community health workers	Promotion and provision of VCT; Psychosocial support; Home visits; Hygiene and nutritional counseling; Adherence support; Ensure accompaniment of patients at the end of the life; Referral to health facilities for medical care; Search for lost to follow up; Food support.	Ensure accompanying at the end of the life; Referral to health facilities for medical care.

*Previously only trained doctors were allowed to perform HIV services. After task shifting, generalist doctors were allowed to use available protocols to perform these tasks, with or without training.

*Abbreviations*: *VCT* voluntary counseling and testing, *ART* Antiretroviral therapy, *PLWHA* People living with HIV/AIDS, *TB* tuberculosis, *OI* opportunistic infections.

### Degree of implementation of the strategy at national level

#### Training process and coverage

Training adopted a cascade model. The training of national trainers started in 2005. By 2008, 61% of the regional trainers had been trained and districts had started training their health staff. Primary health facilities started implementing task shifting in 2007. However, the high turnover made it impossible to achieve sustainable training coverage targets. According to an unpublished survey, 3/5 of health workers were not yet trained as of end of 2011. The duration of the training ranged from three days to one week. There was no difference in training duration by type of cadre. No refresher training was planned.

#### Supportive supervision

There was no plan for supportive supervision that is specific to the task shifting strategy for HIV. In 2006, only 46% of health facilities received at least two routine (not specific to HIV or task shifting) supervision visits per year. In 2007 this went down to 0.65%. In the subsequent two years, none received more than one routine supervision visit per year, if at all [19].

#### Access to VCT, PMTCT and ART services

**Figure 1:**
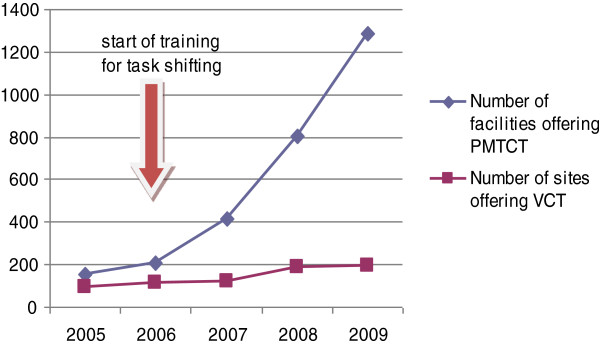
**Access to VCT and PMTCT services (2005–2009).** Source [26].

While sites offering Voluntary Counseling and Testing (VCT) and Prevention of Mother to Child Transmission of HIV/AIDS (PMTCT) services have both increased, it was more so for PMTCT, see Figure 1. In 2009, 1288 (82%) health facilities provided PMTCT services and 192 provided VCT. This is illustrated by the higher percent of pregnant women having access to VCT, through PMTCT services, (around 50%) on average, compared to 16% in 2008 for the general population, Table 2.

**Table 2:** Trends in access to VCT and ART services (2005–2009)

**Indicator**	**2005**	**2006**	**2007**	**2008**	**2009**
Percent of general population having had VCT during last 12 months and been informed about the results	6%	10%	21%	16%*	-
Percent of pregnant women having had VCT during last 12 months and been informed about the results	42%	45%	44%	41%	51%
Number of new patients on ART	8136	12842	17263	21103	26448
% PLWHA who are under ART	25%	54%	37%	43%	53%

Source: [19]. VCT: voluntary counseling and testing. ART: Antiretroviral therapy. PLWHA: People living with HIV/AIDS. * Estimated by source.

The first level of care for initiation of anti-retroviral therapy (ART) and follow up is the district hospital or accredited community centres. The number of patients on ART has more than tripled between 2006–2010. However, 50% of people in need for ART were still uncovered by the end of 2010 [19], see Table 2.

## Methods

### Conceptual framework

The conceptual framework was developed by the study team, which is comprised of multi-disciplinary researchers (FYB, SK, SH, BK and TA) and officials in the Ministry of Health in Burkina Faso (BK). In developing our conceptual framework, we first reviewed the peer-review and grey literature on evaluations of task shifting strategies, including those for non-HIV services, to document all the health systems effects that were evaluated, or argued to be important in understanding the wider range of effects of the strategy on the health system as a whole. All evaluation studies on task shifting or task delegation in LMICs were included. The search was conducted in English and French. No date restriction was set. Search terms included: "delegation of work", "task delegation", "task shifting" in combinations with "outcome and process assessment (health care)", "Program evaluation", or evaluation Studies".

We then considered the findings from the literature review and systematically brainstormed about other possible effects on the health system and its stakeholders that were not captured by the literature, to be explored in our analysis. We did this by systematically considering each of the six building blocks of the health system, and in turn brainstorm on other possible effects of the implementation of the task shifting strategy that could affect, or be affected by, the functions of each of the health systems building blocks. As expected, this was done in several iterations as each effect may have implications on more than one building block. We then drew a list of the key stakeholders that are likely to be influenced by the strategy, either from the supply or demand side and systematically brainstormed on the possible effects of implementing the strategy on each and by each of them. Key stakeholders included: programme managers at government and non-government organizations; trainers and supervisors of health workers; front-line health workers including doctors, nurses, pharmacists, community-based workers etc. working in public or private sectors; beneficiaries, including HIV patients or those interested in their HIV status; local and international funders; and professional associations.

The possible effects that we could identify through both steps are summarized in Table 3. This constituted our basis for exploring the system-wide effects of task shifting for HIV in Burkina Faso and guided our data collection and analysis process. However, this conceptual framework was maintained flexible during the process of this study and the current list of possible effects and the way they are expressed were further refined subsequent to the data collection and analysis.

**Table 3:** Potential intended and unintended effects of task shifting for HIV on the health system as a whole

**Effect**	**Sub-system involved**
***Health outcomes***	
**Better adherence to treatment and reduced loss to follow up**[11].	Service delivery
**Good clinical outcomes** and better survival rates [11].	Service delivery
**Crowding out of other services** where health workers or facilities shift their attention to the new tasks [20,21].	Service delivery; HRH; Health information
***Supply side***	
**Staff burnout** due to workload, for example, due to maldistribution of trained health workers, or additional time to fill HMIS records [20,22].	HRH; Governance; Health information
**Lack of motivation or staff turnover** due to lack of incentives (financial or non-financial) for staff to expand their role [21-23].	HRH; Financing; service delivery; Governance
**Staff turnover** due to lack of career path (e.g., promotion or certification of acquiring the new skills) to address motivation and retention [12].	HRH; Governance
**Low performance** due to selecting health workers (for the training) who are not motivated or interested in the strategy [12].	HRH; Governance; service delivery
**Job satisfaction** due to acquiring new skills and responsibilities.	HRH
**Tension** within health teams about roles and responsibilities and hierarchies, especially with newly developed health cadres [11].	Governance; HRH
**Staff lack of confidence** in performing additional tasks due to insufficient training or supportive supervision.	Governance-HRH-service delivery
**Staff insecurity** when staff do not have legal backing for the additional tasks, impeding them from taking new responsibilities [7,23].	Governance; HRH
**Professional protectionism** due to concerns for being undermined [7,11,23,24]	Governance; HRH
**Staff frustration** due to unavailability of medicines and supplies for diagnostic tests.	Medicines and technology; HRH; Governance; health information
**Cost implications** due to the required supportive supervision and need for new or refresher training to ensure good quality care [7,11,22,25].	Financing; HRH
**Inefficiencies** and poor performance due to over referral, higher use of resources (ordering more lab tests) or lower productivity (longer consultation time) [7,11].	Financing; service delivery; HRH
**Efficiencies** through saving time of senior staff to spend on non-HIV patients or HIV patients with complications and increased utilization at same costs [11].	Financing; HRH; service delivery
**Implications on financing of health care** due to top up of salaries or hiring new cadres.	Financing; HRH
***Demand side***	
**Better services for patients** due to immediate attention, longer consultation including counselling [7].	Service delivery; HRH
**Patient satisfaction** due to reduction in waiting time [11].	Service delivery
**Better access** to HIV services due to services close to home [11].	Governance; Service delivery
**Inequitable access to HIV care** if plans to scale up are not well distributed or do not target remote and rural areas.	Governance; Service delivery
**Implications on financing of health care** due to change in out of pocket expenditures [26].	Financing

**Figure 2:**
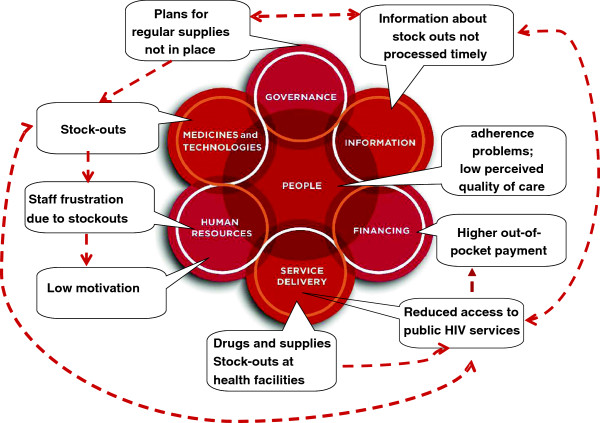
**Illustration of applying a systems thinking approach to evaluating task-shifting: effects of unavailability of medicines and supplies.** Source [16].

As illustrated in Figure 2, which presents the familiar building blocks of the health system [27], components of the system are highly interconnected with each other and what happens in one component often have ripple effects that affect other components in multiple ways [16]. For example, the unavailability of ARV drugs and supplies for HIV will largely depend on existing policies (leadership and governance), which will ensure that information on drug stock-outs are processed timely (health information), avoiding regular stock-outs (medicines and technologies), staff frustration (human resources), inequitable access to care (service delivery) and higher out-of-pocket payments (health financing). Appreciating this reality required us to think systematically and iteratively across the building blocks, considering the possible feedback of each of the effects on the other building blocks. It also meant that using the six building blocks as a framework to “classify” and discuss the possible effects of the strategy on the health system would be impossible since for most effects we could at least identify two or more building blocks concerned (see Table 3). For this reason, we chose not to group the possible effects identified in our conceptual framework by the six building blocks, instead we used the following three categories, as shown in Table 3: health outcomes; supply-side effects and demand-side effects.

### Study design and data sources

We used a case study approach, using qualitative research methods [28]. Data sources included document reviews, and reviews of available data and records, as well as interviews with key informants and health workers. The study was conducted between June and December 2011. Data collection occurred during October and November 2011.

#### Documents and records review

The documents review included reports describing the task shifting strategy and its implementation in Burkina Faso, as well as any progress reports or evaluations available from all stakeholders involved. The objectives of the review were to obtain a clear understanding of: (1) how the task shifting policy was conceived and what were its expected outcomes and targets; (2) how was the intervention implemented throughout the different implementation phases, including the expansion of coverage levels over time; (3) what are the available indicators of its health outcomes; (4) what were the important contextual factors relevant to understanding the impact of task shifting within the settings in which it was implemented and (5) any documented barriers to its implementation to date.

We also reviewed previous data and research on the topic, for example, on patient views and perception on the costs and quality of care for HIV services in Burkina Faso; as well as review of health information systems records and data collected from government sources (at all levels) and partners during the in-depth interviews.

#### In-depth interviews

Semi-structured interviews were conducted with various stakeholders, including health workers, to explore their experience and views with respect to the impact of task shifting, including the negative and un-intended effects on other health services, on human resources more broadly, and the system as a whole. Interview guides were tailored to each type of stakeholder to cover the relevant range of effects discussed in our conceptual framework and allowed for exploring additional effects from the perspective of the respondents through open ended questions, see Table 3.

A convenience sampling approach was used to select the study areas and respondents for the in-depth interviews. Out of the 13 regions in Burkina Faso, three regions were selected for the study (the east, the center and the south-west regions) to represent the different geographic characteristics and HIV prevalence rates in Burkina Faso. HIV prevalence is 0.7%, 1.7% and 3.1% in the east, south west and central regions, respectively [29].

In each region, one district was chosen. In the context of decentralization, districts have the responsibility for organizing primary health services for their population, where around 80% of health facilities are public and the remaining are private or faith-based [30]. HIV services are mainly provided in public and not-for-profit health facilities. In each district, one urban and one rural public health facility were selected for each level of care. Where there are community and/or faith-based health facilities, one of each type was selected. In each facility, the in-charge of the facility was selected for the interview. During the interview with the in-charge of the facility, health workers providing VCT or PMTCT services were identified. Among the health workers who are performing these services we selected one staff from each available cadre/level.

The total sample included 2 regional hospitals, 3 district hospitals, 6 primary health centres, 1 community-based facility and 2 faith-based facilities. Overall 10 urban and 4 rural facilities were included. A total of 19 key informants and 57 health workers were interviewed. Key informants were chosen from groups of Ministry of Health policymakers, development partners, professional associations and community based organizations. Interviews with health workers were conducted with doctors, pharmacists, nurses, midwives, community health workers, and other non-skilled workers from all the various types of public and not-for-profit health facility structures included in our sample.

### Analysis

All interviews were conducted in French. They were audio-recorded and transcribed into a text programme and then converted into Nvivo software for data management and analysis. Interviews were transcribed within a maximum of three days after the interview to avoid recall bias. The initial coding was inspired by the list of possible effects in our conceptual framework and on additional ideas that emerged during the interviews. The quotes included in the text were translated into English by one of the authors and the translations validated by two other authors. All authors are proficient in both English and French.

### Ethical consideration

This study is a sub-component of a larger multi-country study on financing of human resources for health in Burkina Faso, Benin and Niger, for which ethical clearance was obtained from the Ethics Committee for Health Research in Burkina Faso. Respondents participated on a voluntary basis and could withdraw from the study at any time. Informed consent was obtained from all participants and signed consent forms were obtained prior to the interviews. Audiotapes were kept with the principal investigator (FYB) in a locked place, to be destroyed after two years of data collection. Anonymity was maintained to ensure that names and other key information that may identify the respondents are not disclosed, using unique identifier codes kept with FYB. Access to the data was only available to the study investigators.

## Results

The system-wide effects of task shifting on the supply and the demand sides are presented in turn. It is worth noting that some of the conceptualized effects presented in Table 3 were not applicable to our case study (such as lack of career path for new cadres or cost implications due to refresher training or supportive supervision), or were out of the scope of this study (such as impact of the strategy on efficiencies or inefficiencies in health service delivery) so were not addressed below. Others (such as patient satisfaction and cost borne by patients and families for HIV care and treatment) were studied in detail in another study [26] so we used these data in our case study.

### System-wide effects of task shifting on the supply side

#### Staff workload

Health workers' perception of workload was divided among health workers of all cadres. Some perceived an increased workload related to the additional administrative tasks of filling independent forms for HIV services. Others, particularly at the hospital level, have noted that the increase in workload is mainly a function of the reaction of some of their colleagues as this doctor at a district hospital explained: *“the difficulty in our context is that, in structures with nearly 50 health workers, if one receives a specific training, the others tend to refer everyone towards that person. As a result the workload increases”.* However, some respondents, particularly nurses at primary health centres, did not note an increase in workload and explained it by an adaptation and re-organization of services among staff at the facility level to accommodate for the additional services.

#### Staff motivation and performance

Several aspects of health workers motivation have been evoked. The need for carefully choosing health workers to be trained was raised. Some health workers were trained but due to lack of the necessary infrastructure and supplies they were not able to apply what they learnt, which several perceived as a waste and a source of frustration and therefore low motivation. On the other hand, it was also indicated that others who did not have this problem also did not implement the skills they have learnt due to lack of motivation or interest. As this trainer of trainers at national level explained: *“Self-motivation of health workers is very important (…). There are some who only come to have the certificate for their CVs, and after the training they are not self-motivated to perform the new tasks”.*

Another aspect of motivation that was brought up during the interviews was staff morale. Most of the health workers interviewed thought that in the absence of financial incentives, it is important to find other non-financial ways to motivate them. As a nurse in-charge of primary facility suggested, *“even a letter of gratitude and appreciation of their work is comforting”.*

#### Staff Turnover and coping mechanisms

New recruitments or re-assignments of staff due to the general problem of high staff turn-over in Burkina Faso was raised as a concern for the continuity and sustainability of the strategy. The need for staff training was, therefore, always felt. To mitigate this, some facilities have taken their own initiatives to find solutions. For example, using peer-training to ensure that all the personnel are involved in providing the various tasks to avoid the interruption of services in the absence of the trained staff member. This was mainly observed in primary health facilities in rural areas.

#### Job satisfaction

A positive effect that emerged through the interviews is increased sense of responsibility, competence, self-esteem and utility. As a district officer explained, the training and the expansion of tasks and responsibilities have made health workers realize that they can do tasks they previously did not think they can do. This not only gave them confidence in their ability to learn and acquire new skills but also gave them a sense of responsibility for delivering these new services.

In addition, the ability to use the acquired skills for non-HIV services was identified and appreciated by nurses and midwives as a source of satisfaction, where the training on management of opportunistic infections has reinforced their knowledge about other diseases, for example, on recognizing and treating respiratory diseases and dermatologic infections that in Burkina Faso they are authorized to manage given the shortage of doctors at that primary health facilities. It also improved their management of sexually transmitted diseases (STDs), where women attending the antenatal care or family planning clinics were, since they have been trained, systematically examined to exclude STDs, as explained by a midwife in a primary health facility.

#### Relationship between doctors and other staff

Another positive effect of the strategy that emerged from the interviews was a perception of improved relationships between doctors and other health staff. As this doctor at a district hospital explained: *“yes! the workload has decreased, but also it makes you feel trusted! Because when you work with someone and you delegate certain tasks, the person feels appreciated and they do their work well! Consequently our relationship keeps improving".*

#### Staff self-confidence

Despite the increased sense of utility and self-esteem, the majority of respondents, particularly nurses, brought the issue of lack of refresher training and specific supervision for the newly trained in task shifting as limiting factors for optimizing the impact of the strategy. This is exacerbated by the fact that routine supervision has been infrequent as described above. In the words of a nurse in-charge of a primary health facility: *“I would particularly insist on the fact that it is always better when we intend to train people, that we always accompany this training with (…) the necessary technical support. This can be in terms of the necessary supplies, or in terms of refresher training to consolidate knowledge (…), or specific supervision (…), to assess implementation issues on the ground”.*

#### Professional protectionism

Although the current strategy does not authorize nurses to initiate ART, we wanted to explore the views of doctors and nurses on what they think about this possibility. Their views were divided.

Nurses were generally in favour of delegating the initiation of HIV therapy to them. They argued that the shortage of doctors and the fact that nurses are the first line of contact with patients at primary health care facilities, are strong reasons for allowing them to prescribe.

As for doctors, some were in favour of task shifting because this would allow HIV services to be close to home. However, several doctors showed signs of professional protectionism, as illustrated in this statement by a doctor in a regional hospital: *“May be the renewal of prescriptions can be done at the primary facility level, but not the initiation of the treatment. If we come to that, then doctors will have to make their bags and leave Burkina Faso to go to practice elsewhere. I think that is what we should do because if a nurse can do it, doctors will not have their place anymore”.*

#### Staff frustration

The unavailability of medical supplies was perceived as one of the main problems facing the scaling up of the strategy, both for increased access to ARV and also for PMTCT and VCT. This was raised by several respondents as a reason for frustration and lack of motivation as they are unable to apply the skills they acquired during the training or provide the services that the population expects.

Information flows was another reason for frustration, where health workers at the periphery felt that they were not kept up to date with new information released at the national level, for example, on new lines of treatment for HIV/AIDS, something that they only get to know, accidently, through their colleagues in other regions.

### System-wide effects of task shifting from the demand side

#### Patients satisfaction with services received by lower cadres

Interviews with health workers, particularly nurses, at health facilities suggested that patients are generally comfortable with receiving clinical services from lower cadres. As a nurse at the regional hospital explained: *“I can say that it is the paramedics that are in contact 24 /24 with the patient; compared to the doctor who only meets the patients during the consultation (…). Even the patients confuse the nurses and the doctors. They call everyone doctor. Quite often, some patients are more attached to the paramedics than to the doctor”.*

In addition, the task shifting strategy was perceived to have also reinforced the relationship between the patients and health workers as this nurse in-charge of a primary facility explained: *“the fact that you chat with the [pregnant] women when you do the counseling (…) creates a bonding and familiarity between you and the person (…). Often people even come to talk to me about their private life”.*

#### Inequitable access to HIV services and increased financial burden for patients

Understanding the implications of the strategy on equitable access to HIV services was complex. On the one hand, our desk review demonstrated that population in rural areas are disadvantaged, both in terms of equitable access and reduced financial burden for obtaining HIV services, because laboratory testing, ARV initiation and refill are only available in urban areas, where district and regional hospitals are located. Moreover, most VCT centres are located in urban areas. Consequently, the financial burden on households for receiving HIV services remained high as demonstrated in a survey on access to, and costs of, HIV care and treatment in Burkina Faso, where, even if ARV was available free-of-charge, the majority of respondents complained about other costs such as food (91%), and transport (74%) [26].

On the other hand, some respondents, particularly community health workers, noted that for HIV, people prefer services far from home because of fear of stigma. They may, therefore, choose to incur the higher costs of travelling further away from home to mitigate the negative social effects of stigma. It is therefore unclear whether achieving better access to population in rural areas is desirable for them.

## Discussion

This study is the first of its kind attempting to explore the system-wide effects of task shifting in a comprehensive and systematic way. In addition to the traditional measures of impact of task shifting on health outcomes, our study identified 20 possible system-wide effects of the task shifting strategy. Our analytical and conceptual process generated two important methodological observations. First, it illustrated the importance of differentiating between two types of system-wide effects. The first are “effects inherent to the task shifting strategy” itself, such as effects on job satisfaction or better access to health services or better health outcomes. The second are effects due to “health systems barriers”, for example the unavailability of medicines and supplies leading to a series of effects on the various components of the health system, e.g., staff frustration and inequitable access to services. Conceptualizing the possible system-wide effects with these two types of effects in mind guided our brainstorming by ensuring that we systematically and iteratively think through the possible effects and feedback, either positive or negative, generated by each of them.

Second, our conceptual framework demonstrated the complexity and interconnectedness between the different health system building blocks and its actors. While systematically considering each of the six health systems building blocks in thinking through and identifying the possible systems effects was helpful, it was often impossible to attribute any of these effects to only one building block. Working through the possible effects in an iterative and systematic way, with the building blocks in mind, proved a more logical and practical approach to identifying the possible intended and unintended effects of the strategy. In addition, systematically considering the implications of the strategy or its effects on the key health system actors was a powerful approach to identifying and exploring the wider range of relevant effects of the strategy on the system as a whole.

Our findings were comparable to previous studies concerning similar task shifting strategies and settings. For example, findings related to patient satisfaction with care provided by lower cadres [11] and lack of motivation due to lack of financial or non-financial incentives [23]. We also provided new evidence on several health systems effects that were not explored in previous studies on task shifting for HIV, some of which were explored in evaluations of task shifting for other diseases.

Among the new findings that emerged from our study are the positive effects and synergies on health workers’ sense of responsibility and worthiness, their increased job satisfaction emerging from their ability to use the acquired skills in other non-HIV tasks, as well as perceived community appreciation of the services provided at peripheral level and improved patient-provider relationships through better counseling skills, which was the most cited and appreciated outcome of the strategy from the health workers’ perspectives, through its HIV training component. Among the negative or unintended effects is staff frustration due to lack of medicines and supplies or lack of the necessary infrastructure to be able to perform the new tasks. As highlighted above, these two examples are not related to task shifting as a strategy but are a reflection of weaknesses in the underlying system, hence the importance of differentiating between these two types of underlying factors for the observed effects—those inherent to the intervention and those inherent to health systems constraints.

Finally, two of our findings highlight the importance of involving the main stakeholders in designing new health policies or strategies. The first is how to mitigate signs of professional protectionism, where some doctors did not seem to be completely at ease with the possibility of delegating the task of initiating ART to nurses. The second and more complex issue is how to balance the overall objective of making access to HIV services closer to home with patients' fear of stigma, which is driving them as far as possible from home, even when the cost of care was perceived as a huge burden [26]. The latter is not an unusual phenomena and was also observed in Benin [31]. Involving the main stakeholders early on in the design process could have helped identifying solutions to address these concerns and to increase the potential for achieving the desired success and impact. Involving key actors, such as front-line health workers, in implementing and planning for the strategy would have also helped prioritizing needs for health services and justifying the investment in training, supervision, logistics, commodities, and supply chain management with regard to other priorities of the health system.

Our study has several limitations. Our sample size was based on a convenience sample and may be biased towards more urbanized areas. In addition, as the interviews were done at the work place, respondents may have tended to provided positive comments. However, with 76 stakeholders spanning different levels of the health system and including the non-public sectors, these limitations are unlikely to impede the validity of our findings.

## Conclusion

This study adopted a systems thinking approach to explore the system-wide effects of a complex strategy. It demonstrates how the intended and unintended effects of the strategy can be identified and assessed in practice and how the results can be discussed and presented. Our analysis also highlighted the value of adopting a systems thinking approach in designing and implementing new health policies, particularly to mitigate some of the design issues or system bottle-necks that may impede their successful implementation.

## Competing interests

The authors declare that they have no competing interests.

## Authors’ contributions

FYB conducted the literature review and contributed to the initial draft of the conceptual framework and study design; conducted the document reviews, data collection and analysis; and contributed to the interpretation of the results and drafting of the paper. SK, BK and SH contributed to the conceptual framework, the interpretation of the results and provided input to the various drafts of the paper. TA contributed to the literature review, developed the initial draft of the conceptual framework, designed the study, drafted the paper and contributed to the analysis and interpretation of the results. All authors reviewed and approved the final version.

## Pre-publication history

The pre-publication history for this paper can be accessed here:

http://www.biomedcentral.com/1471-2458/13/997/prepub
